# Supplementary material for: A nonrandomized cohort and a randomized study of local control of large hepatocarcinoma by targeting intratumoral lactic acidosis
Source: eLife. 2016 Aug 2;5:e15691. doi: 10.7554/eLife.15691 (PMC4970867; doi:10.7554/eLife.15691)
Supplement: Supplementary file 5. [file elife-15691-supp5.pdf]

Randomized controlled study of bicarbonate-enhanced and conventional transarterial chemoembolization in treatment of hepatocellular carcinoma

Protocol number：ZUSAHZUCI201401
Version：V1.1

Institution in charge of clinical trial：The Second affiliated Hospital, Zhejiang University School of Medicine
Institution proposing the clinical trial：The Second affiliated Hospital, Zhejiang University School of Medicine

Index

Summary of Protocol 3
Abbreviations 6
1 Research background 7
2. The major parameters to assess bicarbonate-enhanced therapeutic efficacy of TACE in treatment of HCC 8
3 Study design 8
3.1 General design 8
3.2 Study population 9
3.2.1 Patient number 9
3.2.2 Inclusion criteria 9
3.2.3 Exclusion criteria 10
3.3 Scheme of study design 10
3.4 Principle of randomization 10
3.5 Dose of chemotherapeutic drugs 10
3.6 Dropout in the study 11
4 Trial procedure 12
5 Follow up after the study 13
6 Adverse effects 13
7 Ethics and written consent form 13
7.1 Ethics 13
7.2 Written consent form 14
7.3 Contingency plan 14
8 Quality control of the trial 14
9 Data processing and storage 14
9.1 CRF 14
9.2 Statistics and analysis 15
10. The period of the trial 15
11 references 15

Summary of Protocol

**Title：**	**Randomized controlled study of bicarbonate-enhanced and conventional transarterial chemoembolization in treatment of hepatocellular carcinoma**
**Purpose：**	To observe if bicarbonate can enhance the therapeutic efficacy of transarterial chemoembolization in treatment of hepatocellular carcinoma
**Method：**	Open randomized controlled study
**Major parameters to observe：**	Tumor response rate to treatment of transarterial chemoembolization with or without bicarbonate
**Secondary parameters to observe：**	Survival times of patients treated with transarterial chemoembolization with or without bicarbonate
**Inclusion criteria：**	1. Hepatocellular carcinoma is confirmed by tissue pathology or clinical diagnosis standard, and does not receive any therapy；<br>2. ECOG score 0-1；<br>3. HCC BCLC grading ： B or C；<br>4. Child-Pugh score prior to therapy： A or B；<br>5. As judged by investigators，the patient can comply with the study protocol；<br>6. Patient voluntarily participates in this study, understands the process of the study, and is willing to sign the written consent form.
**Exclusion criteria：**	1. HCC BCLC grading： 0，A or D；<br>2. Child-Pugh score prior to therapy： D；<br>3. Tumor invades liver and/or portal vein or their branches and there is evident vein artery shunt.
**Dose schedule**	The patients are randomly assigned into bTACE and cTACE groups.

	**cTACE：**<br>TACE was performed through the transfemoral route using a 5-Fr catheter (Shepherd-hook modified Angiographic Catheter, HANACO Medical, Tian Jin, China) that advanced from celiac artery to common hepatic artery, proper hepatic artery, hepatic artery and ultimately to tumor feeding arteries. The tumor feeding arteries are defined by angiography. Then, a coaxial microcatheter (2.8 Fr Marguerite II, ASAHI INTECC GMA CO., LTD, Nagoya, Japan) was selectively inserted through a 5-Fr catheter into the tumor feeding artery, into which, doxorubicin-lipiodol emulsion and oxaliplatin/homocamptothecin was infused. Finally, the artery was embolized with 100-900 μm PVA (Embosphere®, BioSphere Medical, Paris, France) and microcoil (Tornado ®, COOK Medical, USA).<br><br>**bTACE：**<br>TACE was performed through the transfemoral route using a 5-Fr catheter (Shepherd-hook modified Angiographic Catheter, HANACO Medical, Tian Jin, China) that was advanced from celiac artery to common hepatic artery, proper hepatic artery, hepatic artery and ultimately to tumor feeding arteries which are defined by angiography. Then, a coaxial microcatheter (2.8 Fr Marguerite II, ASAHI INTECC GMA CO., LTD, Nagoya, Japan) was selectively inserted through a 5-Fr catheter into the tumor feeding artery, into which, 5% sodium bicarbonate was infused alternatively with doxorubicin-lipiodol emulsion and oxaliplatin/homocamptothecin. Finally, the artery was embolized with 100-900 μm PVA (Embosphere®, BioSphere Medical, Paris, France) and microcoil (Tornado ®, COOK Medical, USA).<br><br>**Follow up：**<br>Tumor response to treatment is assessed by enhanced MRI 30 days after patient is treated by TACE. Patient may receive further TACE treatment if complete

	response is not achieved.<br>For survival time, patients are followed up every 6 months.
**Statistics**	All statistics are carried out using Graphpad Prism software. Statistical test is 2-tailed test, p value less than 0.05 is considered statistically significant.
**Period of the trial**	Start：March, 2014<br>End：October, 2016
**Version**	V1.1

Abbreviations

TACE	Transarterial chemoembolization
cTACE	Conventional Transarterial chemoembolization
bTACE	Bicarbonate-enhanced transarterial chemoembolization
HCC	Hepatocellular carcinoma
DSA	Digital subtraction angiography
ECG	Electrocardiograph

1. Research background

Hepatocellular carcinoma is the sixth most common cancers worldwide and the third leading cause of cancer death[1]. Majority of HCC patients are not eligible for curative therapies (surgical resection and liver transplantation), and TACE is a therapeutic choice for these patients. The objective tumor response to TACE is 35% (range, 16% to 61%, multiple courses of TACE treatment), as systematically reviewed by LIovet and Bruix for the Barcelona-Clinic Liver Cancer Group in 2002 [2]. In the year of 2012, Forner, LIovet, and Bruix summarized that more than 50% of patients had an objective response to TACE [3]. The complete tumor response to TACE is rare (0-4.8%) [4]. Taken together, TACE has reached the therapeutic limits.

In order to increase the therapeutic efficacy of TACE, we implement bicarbonate into TACE procedure, according to the principle that we discovered in the recent years. Lactic acidosis is a common environmental factor many solid tumors. We found out that lactic acidosis are potent in protecting cancer cells exposing metabolic stress. Specifically, we recently[3] identified and confirmed that lactic acidosis was a powerful factor that conferred cancer cells with resistance to glucose deprivation: with lactic acidosis, cancer cells, e.g., 4T1 cell, could survive for 65 days under glucose deprivation, whereas without lactic acidosis, cells died out within 3 days. In addition, under lactic acidosis, cancer cells economically use glucose, exhibits a non-Warburg phenotype, and very limited amount of glucose can support growth of cancer cells, otherwise they die. Very importantly, the effects of lactic acidosis on cancer cells depends on co-presence of proton and lactate, depriving either of which would abolish its roles. When lactic acidosis is neutralized by a base, e.g., bicarbonate, the condition is changed to lactosis (high lactate concentration with a basic pH), which does not have any functions as described above. Likewise, acidosis (low lactate concentration, acidic pH) only offers a weak protection to cancer cells against glucose deprivation. As we know, TACE kills cancer cells through occluding tumor afferent arteries to starve cancer cells and delivering concentrated anticancer drugs locally into tumors. As such, lactic acidosis can antagonize the metabolic stress condition created by TACE and reduce the therapeutic efficacy. Conversely, neutralizing lactic acidosis in HCC by a base such as bicarbonate may significantly enhance the therapeutic efficacy of TACE.

Bicarbonate administration is safe, because the dose used in TACE is well within the safe range. Bicarbonate is safely used in treating metabolic acidosis and tumor lysis syndrome. The safe dose of bicarbonate to treat metabolic acidosis and tumor lysis is 5 mEq/kg of body weight. In this trial, the dose of bicarbonate is far below 5 mEq/kg.

In a pilot study, we have treated 30 patients with HCC with bTACE and found out that bTACE achieved a significantly better therapeutic efficacy than cTACE and that the adverse effects of bTACE and cTACE were similar without statistical significance. To further prove that bTACE is better than cTACE, we designed a randomized controlled study.

2. The major parameters to assess bicarbonate-enhanced therapeutic efficacy of TACE in treatment of HCC

Study purpose：

To compare the therapeutic efficacy between bTACE and cTACE in treatment of HCC。

Major parameters to observe：

Measure the volume of viable residual tumor 30 days after operation using enhanced MRI.

The response to treatment is defined by the viable residual tumor as below: complete response (CR), no obvious viable residuals; near complete response (NCR), viable residuals <10%; partial response (PR), viable residuals >10% but < 50%; stable disease (SD), viable tumor residuals between >50% but ≤100%; and progressive disease (PD), viable tumors > 100%.

3 Study design

3.1 General design

The study is open randomized controlled. The purpose of this study is to evaluate the therapeutic efficacy of bTACE and cTACE in the treatment of HCC. Patients are randomly assigned to test group (bTACE) and control

group (cTACE) in the ratio of 1:1.

3.2 Study population

3.2.1 Patient number

Totally 60 patients would be recruited into the study.

Group	cTACE	bTACE
Number of patients	30	30

3.2.2 Inclusion criteria

1. Hepatocellular carcinoma is confirmed by tissue pathology or in accordance with clinical diagnosis standard, and does not receive any therapy；
2. ECOG score 0-1；
3. HCC BCLC grading ： B or C；
4. Child-Pugh score prior to therapy： A or B；
5. As judged by investigators， the patient can comply with the study protocol；
6. Patient voluntarily participates in this study, understands the process of the study, and is willing to sign the written consent form.

3.2.3 Exclusion criteria

1. HCC BCLC grading： 0、A or D.

2．Child-Pugh score prior to therapy：C.

3. Tumor invades liver and/or portal vein or their branches and there is evident vein artery shunt.

3.3 Scheme of study design

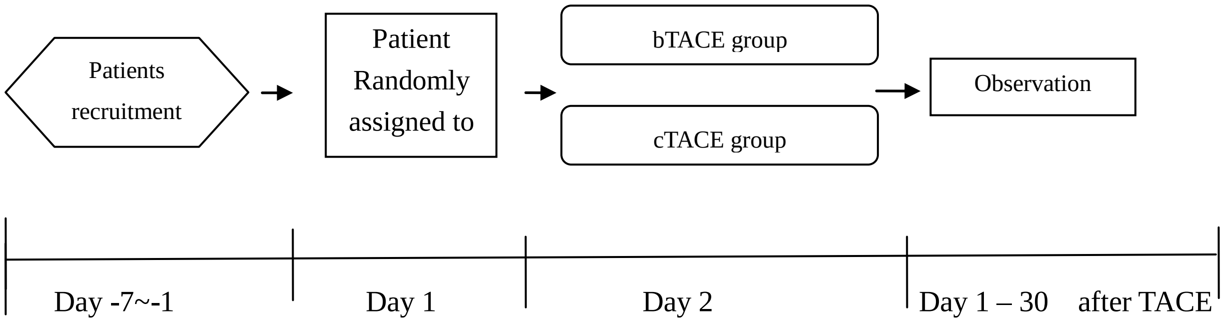

3.4 Principle of randomization

Patients are randomly assigned to bTACE or cTACE according to principle of random number table.

3.5 Dose of chemotherapeutic drugs

<u>cTACE:</u>

Pirarubicin Hydrochloride Injection, 40mg.

Oxaliplatin Injection, 150mg.

Hydroxycamptothecine Injection, 20mg.

Ultra fluid lipiodol.

bTACE：

Pirarubicin Hydrochloride Injection, 40mg.

Oxaliplatin Injection, 150mg.

Hydroxycamptothecine Injection, 20mg.

Ultra fluid lipiodol.

5% sodium bicarbonate injection, 50 – 100 ml

Note:

1. The above drugs are injected into tumor through tumor feeding arteries.
2. The dose of chemotherapeutic drugs, lipiodol, 5% sodium bicarbonate injection are based on the results of DSA and the liver function and also based on the decision of the doctor who operates TACE.

3.6 Dropout in the study

3.6.1 Dropout decided by investigators

Dropout indicates that during study, investigators find that the recruited patients are not suitable for further therapy:

1）severe side effect that does not allow further therapy;

2）Recruited patients do not comply with the study protocol.

3）Recruited patients show progressive disease after therapy.

3.6.2 Dropout decided by patients

According to the written consent, patients have the right to exit from the study. During trial, patients may

be unwilling to continue or may loss of follow up, both of which are considered drop out.

3.6.3 Criteria to terminate the study

Study termination indicates that the study is terminated before completion. The reason to terminate the study can be the following reason:

1) Investigators find that bTACE has a significantly better therapeutic efficacy than cTACE before completing the study. Afterwards, investigators can treat all remaining patients with bTACE.

The purpose to terminate the study is to protect the right of patients. Recruited patients should be informed by a written notice if study is terminated before completion.

3.6.4 The criteria to exclude patients from data analysis

The following patients are excluded from data analysis。

- Not in accordance to inclusion criteria.
- The examination is not in due course.
- Receive other therapy in addition to TACE.
- Progressive disease and intolerable to further TACE therapy.

4. Trial procedure

1) The trial is approved by hospital’s Institutional Review Board.

2) After approval of hospital’s Institutional Review Board, patients are recruited. Recruited patients signed written informed consent. Record the information of recruited patients: demography, history of HCC, allergy, medication history, blood donation, identity card number, communication address, phone number.

3) Recruiting patients: Recruited patients sign written consent form. Prior to TACE, patients were examined serologically (HBsAg, HBs, HBeAg, anti-HBcIgM, and anti-HBcIgG, anti-HCV antibody, anti-HBV antibody)

and by hepatic MRI.

Other examinations include ：

- ECOG score
- Vital sign
- Physical examination
- ECG
- Lab tests: blood routine examination, urine routine examination, feces routine examination, liver & kidney function test, serum AFP, blood clotting test.
- Imaging examination： chest CT scan, liver contrast enhanced MRI scan, liver CT scan.

4) Patients were randomly assigned to bTACE and cTACE groups. The efficacy and safety are evaluated.

5) The examinations during each therapy cycle include:

- ECOG score
- Vital sign
- Physical examination
- ECG
- Lab tests: blood routine examination, urine routine examination, feces routine examination, liver & kidney function test, serum AFP, blood clotting test.
- Imaging examinaiton： chest CT scan, liver contrast enhanced MRI scan, liver CT scan.
- Adverse effect recording.

5. Follow up after the study

After treatment, investigators and patients jointly decide next step therapy. Follow-up visits are scheduled every 6 months.

6. Adverse effects

The following adverse events which might occur during and after TACE procedure were monitored: blood pressure and oxygen saturation during TACE, pain, fever, and signs of liver decomposition after treatment, and biliary system. All the adverse effects should be record in detail.

7. Ethics and written consent form

7.1. Ethics

The trial protocol is approved by the Institutional Review Board (IRB),The Second affiliated Hospital,

Zhejiang University School of Medicine. Any change made in the protocol during trial must be reported and approved by IRB.

7.2. Written consent form

Recruited patients must sign a written consent form. Investigators are obliged to explain to recruited patients the purpose, the benefit, the potential side effect of the therapy. In case that the recruited patient is unable to read the written consent form, in the process of signing written consent form, after patient agree to sign, a witness that can legally represent the patient and the patient shall sign their name on the written consent form on the same date.

7.3. Contingency plan

Operation is performed in the Second Affiliated Hospital, Zhejiang University School of Medicine, which has emergency medical service center. The investigators have experiences in emergency rescue.

8. Quality control of the trial

- Patients sign rewritten consent form.
- Fill out Case Report Form (CRF).
- Keep all data and documents intact.

9. Data processing and storage

9.1. CRF

Investigators must record and sign in time all the related information of recruited patients. CRF are original data and are not allowed to be changed. When some information of CRF is found to be incorrect, the change shall be made with a signature of the investigators and the date.

9.2. Statistics and analysis

9.2.1 Data collection and analysis

To export MRI images of the patients from the database of the Second affiliated Hospital, Zhejiang University School of Medicine as dcm formate, which then import to MIPAV（Medical Image Processing, Analysis, and Visualization）software. The viable and necrotic tumor areas in every layer of MRI image were circled using VOI tool and integrated. Summation of integrated enhanced and nonenhanced area of all slices of a tumor gives the viable and necrotic volume of a tumor. Total tumor volume is the sum of viable and necrotic volume.

9.2.2 Statistical analysis

Statistics (2-tailed test) is carried out using *Graphpad software*. *p value* ≤ 0.05 is considered statistically significant.

9.2.3. Data storage

Investigates must keep data intact.

10. The period of the trial

Start: March, 2014

End: October, 2016

11. References

1. Parkin DM, Bray F, Ferlay J, Pisani P. Global cancer statistics, 2002. CA Cancer J Clin. 2005; **55**(2): 74-108.
2. Llovet JM, Bruix J. Systematic review of randomized trials for unresectable hepatocellular carcinoma: Chemoembolization improves survival. Hepatology. 2003; **37**(2): 429-42.
3. Forner A, Llovet JM, Bruix J. Hepatocellular carcinoma. Lancet. 2012; **379**(9822): 1245-55
4. Jansen MC, van Hillegersberg R, Chamuleau RA, van Delden OM, Gouma DJ, van Gulik TM. Outcome of regional and local ablative therapies for hepatocellular carcinoma: a collective review. Eur J Surg Oncol. 2005; **31**(4): 331-47.
5. Gatenby RA, Gillies RJ. Why do cancers have high aerobic glycolysis? Nature reviews Cancer. 2004; **4**(11): 891-9.
