# Supplementary figures and images for: A nonrandomized cohort and a randomized study of local control of large hepatocarcinoma by targeting intratumoral lactic acidosis
Source: eLife. 2016 Aug 2;5:e15691. doi: 10.7554/eLife.15691 (PMC4970867; doi:10.7554/eLife.15691)

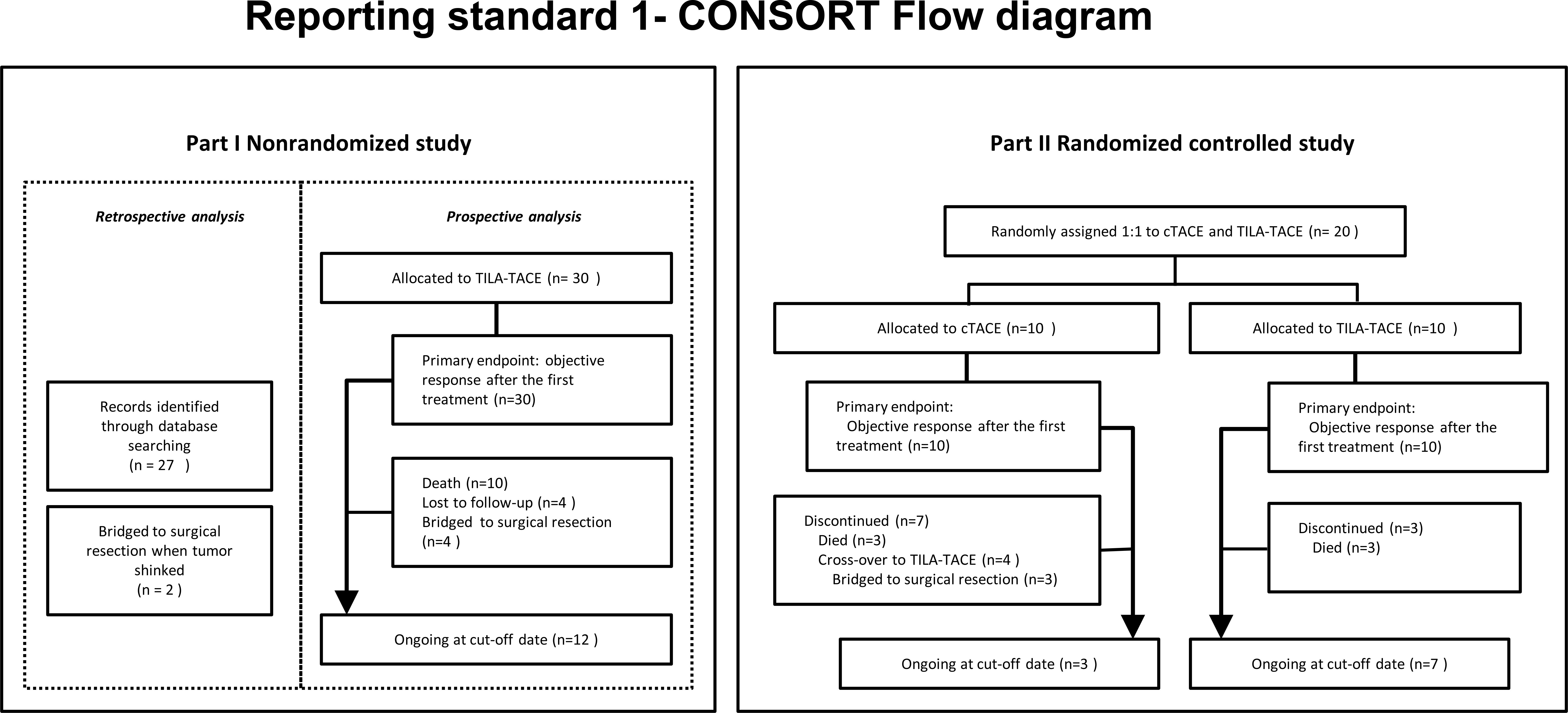

Supplement: Reporting standard 1. [file elife-15691-repstand1.jpg]
